# Role of Ultrasonography in Intraperitoneal Bladder Rupture With Delayed Presentation

**DOI:** 10.7759/cureus.20159

**Published:** 2021-12-04

**Authors:** Ravikanth Reddy

**Affiliations:** 1 Radiodiagnosis, St. John's Hospital, Bengaluru, IND

**Keywords:** resource starved setting, blunt trauma, ultrasonography, child, delayed intraperitoneal bladder rupture

## Abstract

Delayed presentation of intraperitoneal rupture of the urinary bladder in a child after blunt trauma is a rare occurrence. On ultrasonography, diagnosis of bladder injuries has been sparsely reported in the literature. We report a case of intraperitoneal bladder rupture in a four-year-old child and describe the role of ultrasonography in providing a prompt and accurate diagnosis of the entity as mortality increases if surgical repair is delayed. On high-resolution ultrasonography, rent was noted in the urinary bladder dome with fluid collection in the paracolic gutters and between loops of the small bowel. Peritoneocentesis demonstrated urine and the child was taken up for laparoscopic repair of the bladder tear, which was visualized at the bladder dome. Cystogram performed at one week was unremarkable and the post-operative period was uneventful with no complaints at a one-month follow-up.

## Introduction

Around 60% to 85% of all bladder injuries result from blunt trauma to the abdomen but the incidence of intraperitoneal urinary bladder rupture is relatively uncommon and exceedingly rare in children [1]. Delay in the presentation and treatment of the entity increases mortality rate substantially. Clinical symptoms of urinary bladder rupture include the inability to void, lower abdominal pain, and perineal ecchymoses. The hallmark sign of bladder rupture is gross hematuria and has also been associated with microscopic hematuria in 5% of cases [2]. Although computed tomographic cystography is the investigation of choice for diagnosing urinary bladder rupture [3], ultrasonography may have a role in resource-starved settings for providing an early and accurate diagnosis. This is a rare case of delayed presentation of intraperitoneal bladder rupture post-blunt abdominal trauma in the absence of associated pelvic fracture and the report describes the ultrasonography appearances of the entity with the mechanism of injury being sudden compression of the full bladder.

## Case presentation

**Figure 1:**
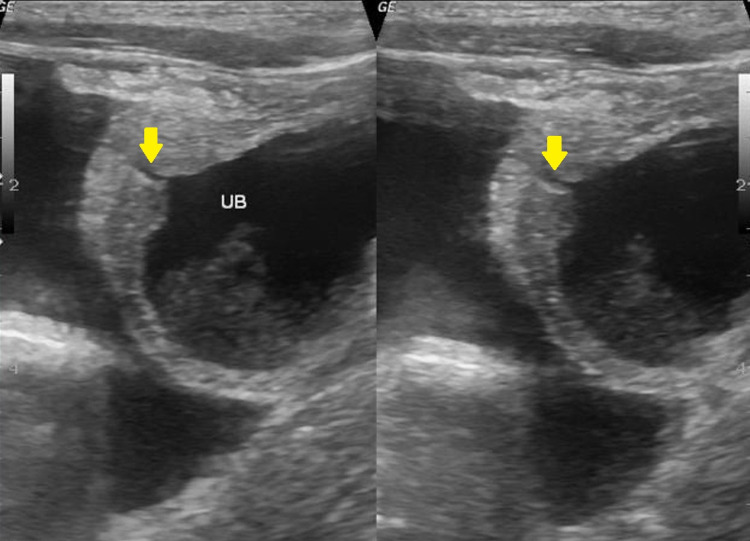
High-resolution ultrasonography image of the pelvis demonstrating intraperitoneal free fluid with evidence of hypoechoic disruption (arrow) involving the dome of the urinary bladder. UB: urinary bladder.

**Figure 2:**
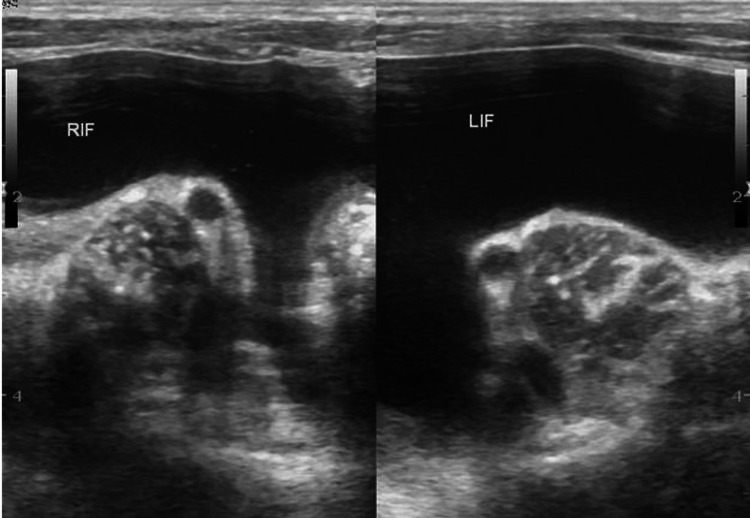
High-resolution ultrasonography image of the pelvis demonstrating fluid collection in the right and left iliac fossae. The margins of the urinary bladder were not separately visualized. RIF: right iliac fossa, LIF: left iliac fossa.

A four-year-old boy presented with complaints of inability to pass urine for 48 hours and insidious onset progressive abdominal distension that developed secondary to trauma to the abdomen while playing. Clinical examination revealed suprapubic tenderness. On day three following the incident of trauma, the child was referred for ultrasonography of the abdomen and pelvis that demonstrated a circumscribed fluid collection measuring 8.0 × 10.0 × 12.0 cm with scattered internal echoes and hyperechoic contents in the dependent portion of the suprapubic region. High-resolution ultrasonography of the pelvis was performed, which demonstrated hypoechoic rent at the dome of the urinary bladder (Figure 1). Margins of the urinary bladder were not separately visualized with fluid collection in the right and left iliac fossae (Figure 2). Color Doppler demonstrated the avascular nature of the cystic lesion. A diagnosis of urinary bladder rupture with evidence of a hyperechoic clot at the dependent portion was made on ultrasonography. Computed tomography (CT) confirmed the findings of ultrasonography as intraperitoneal rupture of the urinary bladder. Peritoneocentesis showed urine, and the child was referred to the department of general surgery for further management. Laparoscopic exploration was performed and a linear tear measuring 1 cm was visualized at the dome of the urinary bladder. Laparoscopic repair was performed and a cystogram performed at one week was normal. The post-operative period was uneventful and the child was discharged after 10 days. There were no complaints of recurrence at a one-month follow-up. The child’s parent has given informed written consent to publish his case and clinical images.

## Discussion

Intraperitoneal rupture of the urinary bladder occurs typically as a result of a direct blow to the already distended bladder and constitutes 15% of major bladder injuries [4]. On ultrasonography, hypoechoic disruption of the urinary bladder wall and free fluid can be visualized in a majority of cases. Distribution of free fluid in the paracolic gutters, mesenteric folds, and around bowel loops is seen in intraperitoneal bladder rupture. Free fluid in the perivesical space is visualized in extraperitoneal bladder rupture and constitutes the majority 85% of major urinary bladder injuries [5]. Differentiation of intraperitoneal or extraperitoneal bladder rupture is important as intraperitoneal rupture requires immediate surgical exploration and extraperitoneal rupture can be managed conservatively. Ultrasonography being a preliminary investigation in trauma to the abdomen and pelvis, its role should be further explored for diagnosing urinary bladder injuries. At the moment, ultrasonography has a limited role with a majority of diagnoses being made during the antenatal period. There is also a scarcity of literature pertaining to the role and findings of ultrasonography in major bladder injuries. High clinical suspicion with visualization of urinary bladder wall disruption and distribution of free fluid in intraperitoneal and extraperitoneal compartments on ultrasonography helps in making a diagnosis of urinary bladder rupture with confidence. On computed tomography (CT), the diagnosis of intraperitoneal or extraperitoneal or combined bladder rupture is made on the basis of extravasated contrast material. However, standard trauma CT protocol remains the investigation of choice in the setting of trauma to the urinary bladder [6].

The standard imaging technique for the diagnosis of bladder injury is retrograde cystography with a reported accuracy of 85%-100% [7], but shows false-negative results, especially in cases with small perforations and inadequate bladder distension with contrast material. In such cases, a CT cystogram is an alternative where even a small amount of contrast extravasation can be detected. Computed tomography (CT) cystogram is performed by instilling water-soluble contrast into the bladder through a Foley catheter (Bard, Covington, GA). The location of extravasated contrast material at CT is useful in differentiating intraperitoneal from extraperitoneal bladder rupture. In intraperitoneal bladder rupture, the CT cystogram demonstrates intraperitoneal contrast material around bowel loops, between mesenteric folds and in the paracolic gutters. In extraperitoneal bladder rupture, the CT cystogram demonstrates extraluminal contrast in the peri-vesical space (molar tooth sign), with contrast extension to the thigh, scrotum, and perineum. Differentiating intraperitoneal bladder rupture from extraperitoneal bladder rupture is crucial for patient management. While intraperitoneal bladder rupture should be corrected surgically, extraperitoneal bladder rupture may be treated conservatively [8]. Extravasation of urine into the intraperitoneal cavity may cause sepsis and further complicate the chances of patient recovery [9]. Although surgical repair is regarded as standard care for intraperitoneal bladder injury, there are multiple cases of intraperitoneal bladder perforation, which were successfully managed conservatively. Nonoperative management options for intraperitoneal bladder perforation secondary to blunt trauma or spontaneous rupture include placement of combined Foley’s catheter and percutaneous peritoneal drain for complete drainage, indwelling transurethral Foley’s catheter alone, or percutaneous peritoneal drain alone [10].

## Conclusions

We present a case of a four-year-old child with blunt trauma to the abdomen and describe the ultrasonography appearances of intraperitoneal urinary bladder rupture with delayed presentation and stresses on the fact that the entity needs to be included in the differential diagnosis of abdomino-pelvic fluid collection, especially in children given the history of trauma. Furthermore, urinary retention should be reviewed with caution in the trauma setting even in the absence of pelvic fractures. This case also highlights the role of ultrasonography in providing an accurate diagnosis of urinary bladder rupture in resource-starved settings. Delayed diagnosis of bladder rupture may be associated with metabolic derangements. Delay in the presentation and treatment may substantially increase mortality. Therefore, early and accurate diagnosis of intraperitoneal bladder rupture with imaging techniques such as high-resolution ultrasonography is imperative for a good prognosis.
